# Pathogenesis and research progress in leukoaraiosis

**DOI:** 10.3389/fnhum.2022.902731

**Published:** 2022-08-19

**Authors:** Lingqi Sun, Lin Hui, Yi Li, Xian Chen, Rong Liu, Ji Ma

**Affiliations:** ^1^Department of Medical Oncology, West China Hospital, Sichuan University, Chengdu, China; ^2^Department of Neurology, Air Force Hospital of the Western Theater of the Chinese People's Liberation Army, Chengdu, China; ^3^Acupuncture and Tuina School, Chengdu University of Traditional Chinese Medicine, Chengdu, China; ^4^Department of Ultrasound Medicine, Air Force Hospital of the Western Theater of the Chinese People's Liberation Army, Chengdu, China

**Keywords:** leukoaraiosis, cerebral small vessel disease, research progress, pathogenesis, clinical features, imaging features

## Abstract

Leukoaraiosis is a common imaging marker of cerebral small vessel disease. In recent years, with the continuous advances in brain imaging technology, the detection rate of leukoaraiosis is higher and its clinical subtypes are gradually gaining attention. Although leukoaraiosis has long been considered an incidental finding with no therapeutic necessity, there is now growing evidence linking it to, among other things, cognitive impairment and a high risk of death after stroke. Due to different research methods, some of the findings are inconsistent and even contradictory. Therefore, a comprehensive and in-depth study of risk factors for leukoaraiosis is of great clinical significance. In this review, we summarize the literature on leukoaraiosis in recent years with the aim of elucidating the disease in terms of various aspects (including pathogenesis, imaging features, and clinical features, etc.).

## Introduction

Leukoaraiosis (LA), also known as white matter hyperintensity (WMH) or white matter lesions (WMLs), is an imaging marker of cerebral small vessel disease (CSVD). Recognition of CSVD dates back to the late 19th century, when Binswanger and his student Alzheimer published the first articles on this topic (Binswanger, 1894; Alzheimer, 1902). However, it now seems likely that the patients they described had vascular changes due to neurosyphilis, and that the original “Binswanger disease” may have described a different pathology than what we currently understand by “small vessel disease” (Grueter and Schulz, 2012). It was only with the availability of modern brain imaging in the 1970s that the discovery of subcortical lesions on brain scans by Hachinski and Awad, respectively, drew attention to these asymptomatic vascular lesions (Awad et al., 1986a,b; Hachinski et al., 1986, 1987).

“Leukoaraiosis” [from the Greek leuko (white) and araios (rarefaction)] is a purely descriptive term, first described by Hachinski, to describe white matter lesions seen on brain scans (Ichikawa et al., 2008). The appearance of LA depends on the imaging method used, but typically it presents as multifocal or diffuse periventricular or subcortical lesions of varying sizes. LA lesions multifocal or diffuse, with indistinct borders are most commonly located in the proximity of the cerebral ventricles or within the semioval center and appear hypodense in comparison to normal white matter on CT images; Magnetic resonance imaging (MRI) is more clear, showing T2-weighted (T2WI) and/or fluid-attenuated inversion recovery sequences (FLAIR) with high signal and T1-weighted imaging (T1WI) with equal signal or low signal, without cystic lesion (Etherton et al., 2016).

LA is common in older adults, especially those with vascular risk factors (Pantoni and Garcia, 1995). In the general population, the prevalence of LA ranged from 11 to 21% in patients around 64 years of age and was as high as 94% in the 82-year-old sample (Debette and Markus, 2010). While it was for a long time regarded as an incidental finding with no therapeutic consequences, there is now increasing evidence that it is associated with specific clinical manifestations such as cognitive decline, gait impairment, mood disorders, urinary dysfunction, and disability (LADIS Study Group, 2011). Given this clinical relevance, prevention and treatment of LA is becoming increasingly important (Bene et al., 2015). With the current aging population, the prevalence of LA is considered rise further. Therefore, In-depth study of LA is increasingly important (Grueter and Schulz, 2012). This paper reviews the progress of LA-related research as follows.

## Pathogenesis

**Figure 1:**
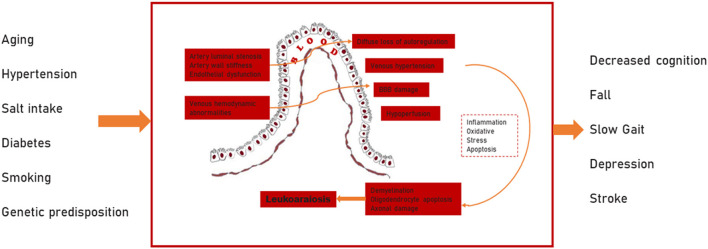
Pathophysiology of leukoaraiosis. The existence of artery luminal stenosis, artery wall stiffness, endothelial dysfunction, and venous drainage limitation due to risk factors such as aging, hypertension, diabetes, and genetic factors, etc. These directly contributes to the diffuse loss of autoregulation, venous hypertension, BBB damage, and hypoperfusion. Thereafter, demyelination, oligodendrocyte apoptosis, and axonal damage occurs in response to stimulation by inflammation, oxidative stress, and apoptosis. The above processes are considered to be the main pathogenic factors of LA (BBB, blood brain barrier).

CSVD, cerebral atherosclerosis, and cerebral amyloid angiopathy, are the most common degenerative vascular diseases in the elderly (Grinberg and Thal, 2010). LA, a subtype of CSVD, is characterized pathologically by pale myelin, demyelination, oligodendrocyte apoptosis, and vacuole formation (Fazekas et al., 1998). Although LA is gaining attention, its pathogenesis is still unclear. It is now generally believed that it is mainly associated with cerebral blood flow autoregulation function, venous collagen deposition, blood-brain barrier (BBB) disruption, invalid neurovascular coupling and genetic factors (Lin et al., 2017; Moretti and Caruso, 2020) (Figure 1).

Several successive studies published by Mok, Poels and Joutel et al. found that hemodynamic alterations may be associated with white matter ischemia (Joutel et al., 2010; Mok et al., 2012; Poels et al., 2012), and impaired dynamic cerebral autoregulation (dCA) is the most common hemodynamic alteration. By assessing cerebral blood flow autoregulation, the dCA process in the middle cerebral artery and posterior cerebral artery bilaterally to represent the dCA of the whole brain, Guo et al. found that the impairment of dCA in CSVD was not limited to unilateral or bilateral effects, but involved the whole brain (Guo et al., 2015). Sclerosis and luminal narrowing of small cerebral vessels caused by chronic hypertension, diabetes or other vascular risk factors may be the main cause of impaired autoregulation of whole brain blood flow (Lin et al., 2017).

Previous studies on the pathogenesis of LA have focused on changes in cerebral arteries rather than veins. Since the concept of periventricular venous collagen deposition was introduced by Moody et al. in 1995, venous collagen remodeling and the influence of the venous system on LA began to receive progressive attention (Moody et al., 1995). Venous ischemia should receive more attention than arterial ischemia. Vascular-derived edema and BBB injury are more common in venous ischemia. In addition, venous ischemia is a long-term, more inert process, and venous ischemia causes pathological features and disease progression that are more similar to LA (Schaller and Graf, 2004). Unlike arterial disease, unilateral obstruction of the jugular venous outflow tract often results in restricted venous drainage in the deep venous system, superficial venous system and watershed areas bilaterally due to venous reflux to the superior sagittal or transverse sinus. This feature makes it more similar to the clinical presentation of bilateral LA (Schaller, 2004). Recently, an increasing number of studies support the association of periventricular venous collagen deposition with LA (Moody et al., 1995; Chung et al., 2011). In an autopsy study of 22 patients aged 60 years or older with arteriovenous differentiation using alkaline phosphatase staining, Moody et al. found that 13 patients had periventricular venous collagen deposition; of these, 10 patients with severe periventricular venous collagen deposition had statistically significant LA (Moody et al., 1995). Although the link between venous collagen disease and LA is unclear, Moody et al. attributed it primarily to a genetic susceptibility (Moody et al., 1995).

LA may also be associated with BBB injury. the entry of secondary serum substances such as serum proteins, complement components and fibrinogen into the brain parenchyma after BBB injury may underlie the pathogenesis of LA (Lin et al., 2017). Using dynamic-enhanced MRI, Starr et al. found that patients with LA had more contrast leakage in arterial penetration areas compared with normal subjects (Starr et al., 2003). Young et al. reduced the bias in selecting different regions of histopathology and their MRI results showed BBB damage in both LA and non-LA regions, thus further illustrating the close association between LA and BBB damage (Young et al., 2008). In addition, capillary pericytes play especially crucial roles in the function of the BBB, which may also underlie the pathogenesis of LA (Uemura et al., 2020). Pericyte ablation leads to breakdown of the BBB in the mouse brain (Nikolakopoulou et al., 2019). Pericytes control protein expression in the tight junctions, their alignment with endothelial cells, and the bulk-flow transcytosis of fluid-filled vesicles across the BBB (Armulik et al., 2010; Bell et al., 2010; Daneman et al., 2010; Quaegebeur et al., 2010). The link between BBB and LA has been confirmed by human and animal studies, and with the development of imaging techniques, BBB damage has been identified as a cause of LA. However, there is also a study claiming that BBB damage is not associated with LA (Wahlund and Bronge, 2000). More studies with less bias and more plausibility are still needed to confirm the association between LA and BBB.

Recent studies found that the occurrence of LA may also be associated with invalid neurovascular coupling. As far as we know, endothelium distress can potentiate the flow dysregulation and lead to microglia activation, chronic hypoxia and hypoperfusion, vessel-tone dysregulation, altered astrocytes, and pericytes functioning blood-brain barrier disruption, which may be the pathogenetic basis of LA. The apparent consequence (or a first event, too) is the macroscopic alteration of the neurovascular coupling (Moretti and Caruso, 2020). This system has many complex functions„ but it seems likely to exert the drainage work of the brain. Therefore, modification of this system produces deleterious effects, whose results are an accumulation of catabolites and toxic substances, together with a pronounced neural starvation (Abbott et al., 2018; Sweeney et al., 2018). In LA, the pathological cascade of events, which occurs as a consequence of the inflammatory/obstructive/stagnation-induced process, determines a decrease of the vascular tone, with a release of the blood-brain barrier permeability, with a loss of the internal vascular remodeling and with major vascular rarefactions. As a result, hypo-perfusion at rest occurs in the brain and it is associated with a diminishment of the neurovascular coupling (Wardlaw et al., 2013; Liu et al., 2017). LA could also affect the integrity of the medial cholinergic pathway, for the hypoperfusion preferred localization, in the deep white matter capsule, or, due to the multiple lacunar infarcts, the basal forebrain cholinergic bundle could be deafferentated from the tubero-mamillary tracts (Zhan et al., 1994; Román, 2004; Bohnen et al., 2009). These aspects affect the normally-accurate cerebral flow regulation and they can further disturb the “retrograde vasodilatation system” with necessary consequences in neurovascular coupling (Ahtiluoto et al., 2010). On the other hand, LA usually implies a reduced metabolic rate of oxygen (estimated of about 35% in white matter) (Yao et al., 1990; Furuta et al., 1991); metabolic incongruity between the brain oxygen supply and its consumption has been described in LA, which determines an altered neurovascular coupling and altered vasomotor reactivity (Tak et al., 2011; Caruso et al., 2019).

With the development of genetic technology, research on the pathogenesis of LA has gradually begun to focus on genetic factors, which may play an important role in the development of LA by as much as 55–80%(Atwood et al., 2004). Research on genetic susceptibility to LA has been divided into two main categories: candidate gene association studies (CGAS) and genome-wide association studies (GWAS). Genome-wide linkage analyses of LA in the past decades have found that LA links to chromosomes 4, 5, 1 and 11 (Turner et al., 2005; Destefano et al., 2006; Kochunov et al., 2009). Studies on CGAS, GWAS and gene expression suggest that neuroimmunity, inflammation, oxidative stress and apoptosis may be involved in the formation of LA (Lin et al., 2017). Some animal studies on LA also suggest that genetic factors are involved in its pathogenesis (Lin et al., 2001; Lan et al., 2015). A small number of LA genetic linkage analysis studies suggest that some certain specific genes are closely associated with LA, but their exact loci have not been identified (Lin et al., 2017). Notably, there were no reproducible associations between the genes obtained from GWAS and LA. Moreover, some of the associations presented in CGAS were not identical to the results in GWAS (Fernandez-Cadenas et al., 2011).

## Neuroimaging and diagnosis

### CT and MRI

Although the term leukoaraiosis was introduced based on CT images, lesions of LA are more clear and well defined on MRI scans, especially using T2WI and FLAIR sequences. LA appears as a low attenuation area on CT and as a high signal area on T2WI or FLAIR. MRI is more sensitive than CT for the detection of small lesions (O'sullivan, 2008). Thus LA can be detected earlier on MRI, but not always in conjunction with neurological deficits. In contrast, a clearer correlation between imaging manifestations and neurological deficits can be observed on CT, as lesions found on CT reflect more severe neurodegenerative degeneration (Zagrajek and Pokryszko-Dragan, 2005). Gradient echo sequences (GRE) or more sensitive MRI magnetic susceptibility weighted imaging (SWI) can demonstrate the presence of cerebral microhemorrhages that cannot be distinguished from ischemic small vessel disease on CT and that are often characteristic of hypertensive small vessel disease and cerebral amyloid angiopathy (Yuan et al., 2018). LA can be divided into periventricular LA and deep/subcortical LA based on MRI features. periventricular white matter receives blood supply mainly through long penetrating branches and extraventricular isolated vessels, and these features make periventricular LA attributable to ischemia and ependymal layer injury (Rowbotham and Little, 1965). The subcortical white matter receives its blood supply mainly through short branch arteries from long penetrating branches, a feature that makes it vulnerable to long-term chronic ischemic and hypoxic injury (Rowbotham and Little, 1965). Usually, however, this disease does not damage the tangentially moving white matter fibers (i.e., U-shaped fibers) at the junction of the gray and white matter.

The progression of LA tends to follow a general pattern. Periventricular lesions initially occur apically in the lateral ventricular horns, but as the disease progresses, its severity may extend to the periventricular area. Deep white matter lesions usually occur first in the frontal lobe, with subsequent involvement of the parieto-occipital lobe and, rarely, in the brainstem and basal ganglia regions (Ornello et al., 2018). This lesion rarely involves the temporal lobe, an important feature that distinguishes it from the autosomal dominant small vessel arterial disease, CADASIL (Mijajlovic et al., 2011). They are not apparent in mild LA, but as disease severity increases, these lesions fuse together and eventually involve the entire region diffusely.

The severity grading of LA relies mainly on the judgment of the observer and is therefore more subjective. To increase the objectivity of the ratings, different rating scales have been developed, which can vary in complexity and ease of use. Among the many scales used to grade the severity of CT, the scale developed by Swieten et al. in 1992 has been widely used in clinical (Marek et al., 2018). With the development of magnetic resonance imaging techniques, several MRI-based scales have been proposed to assess the degree of white matter involvement. Notably, the scale developed by Fazekas et al. in 1987 is the most commonly used in clinical practice (Grueter and Schulz, 2012; Marek et al., 2018). This scale assesses the involvement of periventricular white matter and deep white matter. The periventricular white matter was rated as follows: 0-no lesion, 1-“caps” or pencil-thin lining, 2-smooth “halo”, 3-irregular periventricular signal extending to deep white matter. And the deep white matter is graded as follows: 0—no lesion, 1—punctate lesion, 2—beginning of fusion, 3—extensive areas of fusion (Scheltens et al., 1998). It is difficult to determine which scale is the most accurate. MRI is more refined for LA analysis because of better tissue resolution. For simple scales, assessing the severity of LA can be difficult due to the subjective nature of the assessment and the experience of the assessor. On the other hand, the use of complex and time-consuming scales is less feasible in a busy clinical setting. As a result, LA is often simply classified as “none,” “mild,” “moderate,” or “severe.

### Transcranial doppler ultrasound

Efforts should be directed to find portable and reliable screening diagnostic tools that may help identify candidates for MRI screening in remote areas where MRI is not available. Previous studies have applied transcranial Doppler ultrasound (TCD) to explore the relationship between cerebral hemodynamics and brain lesions attributed to small vessel disease in cognitive disorders (Heliopoulos et al., 2012; Mok et al., 2012; Malojcic et al., 2017). The rationale is that early changes in the intracranial blood vessel wall can be reliably identified by ultrasound techniques, which allow to detect even minimal or subclinical changes (Demarin and Morovic, 2014). TCD, through the evaluation of the mean blood flow velocity (MBFv) and the Gosling's Pulsatility Index (PI), is able to assess the cerebral hemodynamics of the main cerebral arteries. While MBFv is a relative measure of the arterial perfusion integrity, PI reflects the resistance of the small vessels and the intracranial compliance (Baumgartner, 2006; Wagshul et al., 2011). Therefore, TCD is an inexpensive and feasible alternative to evaluate the cerebral hemodynamics, the arterial perfusion integrity, and the intracranial small vessel compliance (Wagshul et al., 2011; Brutto et al., 2015; Vinciguerra and Bosel, 2017).

A recent TCD study evaluated cerebral hemodynamics in patients with late-life depression and subcortical ischemic vascular disease, shows that a diffuse cerebrovascular pathology likely arising from the small vessels and then extending to larger arteries (Puglisi et al., 2018). Vinciguerra et al. assessed indices of cerebral blood flow velocity in vascular cognitive impairment-no dementia patients and to correlate TCD changes with neuropsychological scores and white matter lesions severity. The results show that specific measures of cerebral perfusion and vascular resistance were significantly associated with white matter lesions and executive performance in patients with vascular cognitive impairment-no dementia. These changes may be considered as the TCD correlate of vascular cognitive impairment-no dementia due to microcirculation pathology (Vinciguerra et al., 2019). Hospital-based studies in high-risk or stroke patients have found an association between the PI of intracranial arteries and LA of presumed vascular origin (Xiong et al., 2013)^.^ These findings suggest TCD represents a valuable tool in the early detection, assessment, and management of LA patients at risk for dementia.

However, a recent research shows that in a representative sample of older adults living in a rural Latin American population, the PI of major intracranial arteries do not correlate with LA severity after adjusting for confounding variables (Brutto et al., 2015). A high PI may not only reflect distal cerebrovascular resistance (and thus, LA) but may also occur as the result of large artery stiffness or other hemodynamic factors (Webb et al., 2012). Because of its complex nature, PI is not useful to assess LA prevalence and should not be used alone as a proxy for LA (Brutto et al., 2015). However, Further cooperative studies are still needed to settle the role of TCD in mass screening of MRI candidates for LA assessment.

### Transcranial magnetic stimulation

There is a need to find suitable biomarkers for the early stages of LA that could be tested non-invasively and be cost-effective toward the development and serial assessment of novel treatment strategies, due to its relationship with stroke, cognitive impairment, mood and behavioral, urinary disturbances, and motor function disturbance, typically vascular Parkinsonism and vascular cognitive impairment (Wardlaw et al., 2013; Korczyn, 2015; Di Lazzaro et al., 2021). Transcranial magnetic stimulation (TMS) is a powerful tool to probe *in vivo* brain circuits, as it allows to assess several cortical properties, enabling the identification of potential markers of the pathophysiology and predictors of cognitive decline; moreover, applied repetitively, TMS holds promise as a potential therapeutic intervention (Di Lazzaro et al., 2021). This property has led to many studies investigating the relation of dysfunction of intracortical circuits or cortical plasticity with specific clinical characteristics.

Cholinergic function was initially tested by means of TMS to distinguish dementias with neuropathological evidence of alteration of cholinergic pathways, such as Alzheimer's disease (AD) and Dementia with Lewy bodies (Di Lazzaro et al., 2002, 2007), from non-cholinergic forms of dementia, such as frontotemporal dementia (FTD) (Di Lazzaro et al., 2006). Testing of cortical connectivity can also be used to find hallmarks of sensorimotor cortical dysfunction in AD (Ferreri et al., 2016). Moreover, synaptic dysfunction disrupting the physiological process of synaptic potentiation after repeated activation is emerging as another possible neurophysiological marker of AD (Di Lorenzo et al., 2016; Motta et al., 2018). While a single TMS measure offers low specificity, the use of a panel of measures and/or neurophysiological index can support the clinical diagnosis and predict progression (Di Lazzaro et al., 2021).

In addition, TMS represents a powerful mean to probe *in vivo* the synaptic function and plasticity at different disease stages even in the very early phases when there are no structural changes, and at the same time it may be used as a therapeutic tool to modulate maladaptive plasticity in patients (Di Lazzaro et al., 2021). So far, only repetitive TMS (rTMS) over the left dorsolateral prefrontal cortex and multisite rTMS associated with cognitive training have been shown to be, respectively, possibly (Level C of evidence) and probably (Level B of evidence) effective to improve cognition, apathy, memory, and language in AD patients, especially at a mild/early stage of the disease (Bentwich et al., 2011; Fiorenzato et al., 2017). The clinical use of this type of treatment warrants the combination of brain imaging techniques and/or electrophysiological tools to elucidate neurobiological effects of neurostimulation and to optimally tailor rTMS treatment protocols in individual patients or specific patient subgroups with dementia or mild cognitive impairment (Di Lazzaro et al., 2021). rTMS worked in synergy with medication at least in alleviating behavioral symptoms present in AD, suggesting positive interactions between modulatory after-effects of rTMS and pharmacological medication (Wu et al., 2015). However, it should be noted that the very nature of concomitant rTMS and medication use is not well understood and further research is needed in this area (Hunter et al., 2019). Finally, some electroceutical therapies such as transcranial direct current stimulation (tDCS) might also modulate alterations in excitatory/inhibitory balance in dementia with Lewy bodies, such as in the treatment of visual hallucinations (Di Lazzaro et al., 2021). However, the efficacy of tDCS have been equivocal in this regard (Elder et al., 2016, 2019).

Taken together, TMS, coupled with current biomarkers, could ease the detection of neural degeneration in a phase when it is still modifiable. Indeed, TMS could be able to capture subtle changes years before the conversion to manifest dementia. An early, accurate diagnosis of dementia will likely be fundamental when designing trials of disease modifying drugs (Di Lazzaro et al., 2021).

## Epidemiology

The prevalence of LA varied widely across studies, ranging from 5.3 to 95%(Dufouil et al., 2001; Wardlaw, 2001). This large variability may be due to methodological differences between studies, such as differences in imaging assessment methods, risk factors, and study populations. It is difficult to assess whether prevalence of LA has changed over time due to its prevalence varies so much. Better image quality and more sensitive brain imaging techniques have higher diagnostic rates for LA, but population aging may be the real reason for the increased prevalence of LA (Park et al., 2007). However, today there is a growing awareness of LA risk factors, and prevention and control of hypertension may reduce the incidence of LA. Thus, the apparent increase in LA incidence due to improved quality of brain imaging may have been offset by the actual decrease due to improved blood pressure control (Lovelock et al., 2007).

## Risk factors

**Figure 2:**
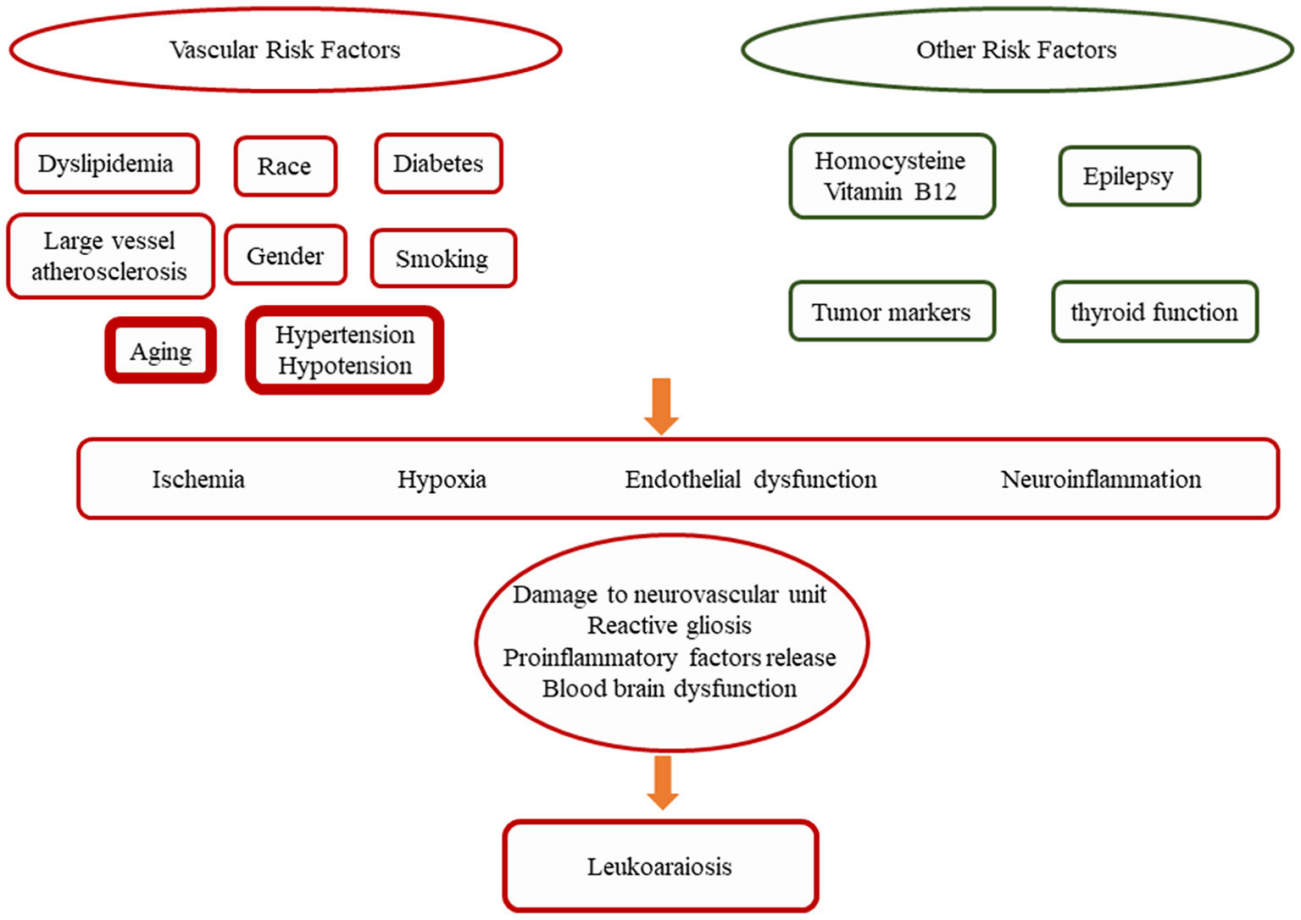
A synopsis of the possible risk factors conditioning the progression of LA. Among them, age, hypertension and hypotension are considered to be the most important risk factors for LA (Grueter and Schulz, 2012).

Although the pathogenesis of LA is not fully understood, it is generally accepted that its incidence increases with age, especially in people over 60 years of age (Grueter and Schulz, 2012) (Figure 2). Other widely accepted risk factors include female, hypertension, hypotension, heart disease, type 2 diabetes, abdominal obesity, hyperlipidemia, hyperhomocysteinemia (HHcy), carotid stenosis, history of stroke, smoking, alcohol abuse, and chronic kidney disease and etc. (Gottesman et al., 2010; Etherton et al., 2016; Marek et al., 2018; Caruso et al., 2019). Still controversial risk factors include epilepsy, tumor markers, and thyroid function in relation to LA development (Ferlazzo et al., 2016; Seo et al., 2019; Son et al., 2020). Due to the differences in research methods, some of the studies have contradictory results.

### Age

Advanced age may be the most important risk factor for the development of LA (Grueter and Schulz, 2012). This is why LA is often referred to as “age-related leukoaraiosis”. Although LA is a pathological phenomenon, it may also be part of the normal aging process to some extent. Although LA is a pathological phenomenon, it may also be part of the normal aging process to some extent. However, it is not clear at what age white matter lesions begin to develop and there is a lack of precise data on the extent of disease that can be considered “normal” at a certain age (Pantoni and Garcia, 1997; Hopkins et al., 2006). Most studies suggest that at least some white matter lesions are predictable after age 50–65 (Srikanth et al., 2009). LA is undoubtedly more common in the elderly and has a higher incidence with increasing age (Grueter and Schulz, 2012).

### Gender

Studies on the relationship between gender and LA prevalence have shown conflicting results. Some findings find a higher prevalence of LA in women, while others suggest that men are at higher risk of developing LA (Grueter and Schulz, 2012). The differences in the results of these studies may be due in part to differences in the characteristics of the study populations, as well as the influence of a number of other confounding factors. For example, the study that found that men were at higher risk was in the Japanese study population, while the other two studies were in the U.S. population (Park et al., 2007). In addition, Simoni et al. suggested that there may also be differences in age or hypertension prevalence between men and women, suggesting a significant gender difference in LA prevalence (Simoni et al., 2010).

### Race

One reason why LA appears to be more prevalent in Afro-Caribbean populations than in Caucasian populations may be due to the higher prevalence of hypertension in Afro-Caribbeans (Gottesman et al., 2010), who may have more severe hypertension and tend to have poorer control of their blood pressure than Caucasians. Also, differences in genetic factors between Afro-Caribbean and Caucasian populations may modify the effects of hypertension on LA development (Meadows et al., 2009).

### Hypertension

Hypertension is closely related to LA and may be the most important controllable risk factor for LA (Marek et al., 2018). Hypertension promotes the underlying mechanism of LA, promoting micro-athero-matosis, which can rapidly lead to stenosis, or different degrees of vessel occlusion until the complete lumen occlusion, inducing ischemia and brain damage (Laurent et al., 2003). On the other hand, chronic hypertension and hypotension alter cerebral blood flow autoregulation and may affect the autoregulatory range (Caruso et al., 2019). Relevant studies have clarified that hypertension plays an important role in the development of LA, with elevated systolic and diastolic blood pressure associated with it (Pantoni and Garcia, 1997; Birns et al., 2009; Simoni et al., 2010). Chronic hypertension has been shown to accelerate amyloid deposition, blood-brain barrier (BBB) dysfunction, microglial cells activation, and subsequent neuronal loss and development of LA (Kruyer et al., 2015). No clear threshold has been found for blood pressure for the development of disease, and the relationship is continuous. In addition to the absolute value of blood pressure, abnormal diurnal blood pressure fluctuations may also be associated with LA (Etherton et al., 2016).

### Hypotension

Hypertension might be a historical problem for LA, but hypotension might be even more dangerous by comparison. Vascular aging is associated with changes in the mechanical and structural properties of vessel walls, which leads to the loss of arterial elasticity reducing arterial compliance (Jani and Rajkumar, 2006). This induces an alteration in autoregulatory capabilities of cerebral arteries, responsible of cerebral perfusion at a constant rate of blood pressure. In this situation, the brain may be more vulnerable to ischemic insults when systemic blood pressure dips below a critical threshold for maintaining perfusion (Torre, 2002). Orthostatic hypotension is arbitrarily defined as a fall in systolic BP of 20 mmHg, or a fall in DBP of 10 mmHg on standing, but when associated with symptoms suggestive of cerebral hypoperfusion, an even smaller drop in BP may be of equal importance (Mathias and Kimber, 1999). Several epidemiological studies have also described low blood pressure, especially in later life as a risk factor for the development of LA, pointing to the potential risk of over treating hypertension (Verhaaren et al., 2013; Venkat et al., 2015). Several studies have shown that sudden postural hypotension can cause relatively prolonged changes in brain perfusion and may contribute to cause small lacunar events or contribute to white matter alterations and LA (Londos et al., 2000; Kaufmann and Biaggioni, 2003). These aspects determine the sensitivity of patients with LA to hypotension and each clinical condition that may lead to hypotension might accelerate underlying disease processes (Caruso et al., 2019).

### Diabetes

Studies on the effects of diabetes on LA have shown conflicting results. Some studies suggest an association between LA and diabetes, especially with periventricular lesions (Bene et al., 2015). Similarly, elevated fasting glucose has been found to be associated with LA. In a study, Anan et al. found that insulin levels were significantly higher in patients with LA (Anan et al., 2009). The above data suggest that insulin resistance is a risk factor for LA. Nevertheless, the strength of this association and the pathological mechanisms involved are not clear. There are also studies suggesting no significant relationship between LA and diabetes (Bene et al., 2015).

### Dyslipidemia

Dyslipidemia is an important risk factor for macrovascular disease and whether it is also a risk factor for small vessel disease has not been established. park, Anan and Kocer et al. have successively shown that both low levels of HDL and hypertriglyceridemia may increase the risk of developing LA (Kocer et al., 2005; Park et al., 2007; Anan et al., 2009). However, the study by Padovani and Streifler et al. failed to find a correlation between dyslipidemia and LA (Streifler et al., 1995; Padovani et al., 1997).

### Smoking

Whether smoking history influences the development of LA is unclear. Park and Dijk et al. found that smoking history and LA appear to be correlated (Park et al., 2007; Dijk et al., 2008), but the study by Padovani and Streifler et al. did not find a difference in the prevalence of LA between smokers and non-smokers (Streifler et al., 1995; Padovani et al., 1997).

### Large artery atherosclerosis

Since the underlying pathological process of LA is considered to be a small vessel lesion, large vessel atherosclerosis and LA are not necessarily related. Breteler, Ohmine and Manolio et al. found an association between atherosclerotic disease and LA, which may be caused by two different mechanisms (Breteler et al., 1994; Manolio et al., 1999; Ohmine et al., 2008): First, the narrowing of blood vessels caused by atherosclerosis reduces the blood flow to the brain, which increases the risk of chronic ischemia in the brain and also increases the risk of LA (Etherton et al., 2016). Second, atherosclerotic disease and LA share a number of risk factors and therefore may occur together and affect each other (Etherton et al., 2016).

### Vitamin B12 and homocysteine

Homocysteine (Hcy) is a sulfur-containing amino acid generated during methionine metabolism, accumulation of which may be caused by genetic defects or the deficit of vitamin B12 and folate. Hcy has many roles, the most important being the active participation in the transmethylation reactions, fundamental for the brain (Moretti et al., 2021). Hcy accumulation could interfere with endothelium dysregulation, favor oxidative damage, and promote neuroinflammation and neurodegenerative processes, all of which occur in LA (Surtees et al., 1997; Rutten-Jacobs et al., 2016; Piao et al., 2018; Moretti, 2019).

While many studies focused on thrombosis and HHcy, HHcy and coronary disease, stroke, and major vessel disease, few data are available on HHcy and vascular and neurodegeneration because LA in the brain is a relatively recent entity (Moretti et al., 2021). HHcy exerts essential alteration in the LA pattern. HHcy induces an increase of Abeta 1–40 toxicity on the smooth muscle cells of the brain's small arteries, where cerebral amyloid depositions occur, transforming the event into cerebral amyloid angiopathy, a constant finding in overt LA condition (Zhao et al., 2013; Caruso et al., 2019; Moretti and Caruso, 2019). HHcy also promotes a constant enhancement of microglia activation, inducing the sustained pro-inflammatory status observed in LA (Moretti et al., 2021).

The correlation between low vitamin B12 levels and LA, especially with periventricular lesions, has been reported in a study by Pieters and de Lau et al. (De Lau et al., 2009; Pieters et al., 2009). A very recent study showed a dose-independent relationship between the plasma Hcy levels and the development of LA (Ji et al., 2020). The study needs to be confirmed in a much larger number of patients. However, although Wright and Hassan et al. showed a correlation between low vitamin B12 levels and the resulting HHcy and LA, there are still not enough studies to prove that vitamin B12 treatment or lowering Hcy levels in the body can improve LA or slow its progression (Hassan et al., 2004; Wright et al., 2005). Furthermore, cellular hypomethylation caused by build-up of S-adenosylhomocysteine (AdoHcy) also contributes to the molecular basis of Hcy-induced vascular toxicity, a mechanism that has merited our attention in particular. AdoHcy is the metabolic precursor of Hcy, which accumulates in the setting of HHcy and is a negative regulator of most cell methyltransferases (Esse et al., 2019). More importantly, AdoHcy has been claimed to be a better indicator of LA than Hcy (Kerins et al., 2001; Valli et al., 2008; Xiao et al., 2015).

Definition of the different roles of Hcy at the different cellular levels, promotion of the confluency of altered white matter areas, and times of the development of SVD in the brain may provide hints as to the modulation of Hcy to prevent disease (Moretti et al., 2021).

### Epilepsy

Although the relationship between stroke and epilepsy has been extensively studied, there is still less attention paid to LA and epilepsy. Maxwell and Okroglic et al. suggested a mutual facilitation between late-onset epilepsy and LA occurrence in the elderly (Maxwell et al., 2013; Okroglic et al., 2013); however, the results of Gasparini et al. suggested that the association may be contingent (Gasparini et al., 2015). Therefore, further animal and clinical studies are needed to clarify and explain whether LA is merely an incidental imaging finding in patients with epilepsy or whether it does play a role in the pathogenesis of epilepsy.

### Tumor markers

Two studies in Korean population by Son and Seo et al. suggested that CA199 and CEA levels of tumor markers were positively correlated with LA (Seo et al., 2019; Son et al., 2020). The correlation remained significant after controlling for confounding factors including age, sex, body mass index, and lifestyle. However, the exact mechanism of the effect is unknown, and it is speculated that the possible causes are endothelial dysfunction, insulin resistance, as CA199 and CEA were found to be elevated in patients with non-malignant atherosclerosis and diabetes mellitus. However, the above study was limited to a cross-sectional study in the Korean population.

### Thyroid function

Available data suggest a correlation between changes in thyroid function and the development of LA. Leonards and Zhang et al. found that thyroid stimulating hormone and subclinical hypothyroidism were positively associated with LA (Leonards et al., 2014; Zhang et al., 2017). The exact mechanism responsible for this interplay is unclear, and the aforementioned study attributes it to the effect of thyroid hormones on microvascular endothelial function and the systemic inflammatory response due to LA.

## Clinical manifestation

### Cognitive decline and dementia

Data from early cross-sectional studies have indicated a possible association between LA and cognitive impairment (Pantoni and Inzitari, 2005). Cognitive decline caused by LA presents with executive dysfunctions, attention and memory decline, set-shifting disabilities, slower speed of information processing, decline of verbal fluency, and delayed recall. On the behavior area, symptoms showed apathic, mood disorder, depression and daily living disability (Pantoni, 2010; de Laat et al., 2011; Del Bene et al., 2013). Among others, some clinical features include sleep disorders, vertigo, tinnitus, and hearing disorder (Li et al., 2018). White matter lesions are a predictor of cognitive decline and dementia and there is a correlation between progression of white matter lesion load and decline in cognitive performance (Schmidt et al., 2007; Jokinen et al., 2009). Therefore, LA are an important substrate for cognitive impairment (Pantoni, 2010). However, LA are not associated with global cognitive decline unless other lesions are also present, and they should not be considered as an indicator of dementia (Frisoni et al., 2007).

LA is today thought to be among the main causes of vascular cognitive impairment (Pantoni, 2010). vascular cognitive impairment associated with LA is thought to be a progressive condition from normal cognitive status to frank dementia (Pantoni et al., 2009). As well as cognitive disorders, the clinical characteristics of vascular cognitive impairment associated with LA are gait, mood and behavioral, and urinary disturbances (Pantoni, 2010). In the early phases, these disturbances can be mild and loosely associated. The final stage is one in which the patient fits the criteria for dementia (i.e., cognitive deficits have a clear and relevant effect on the functional status), gait is very impaired with many patients almost unable to walk and having frequent falls, mood is altered with prominent depressive symptoms or apathy, and urinary incontinence is present (Pantoni, 2010).

### Age-related disability

Because white matter lesions are not only associated with cognitive disorders but also with gait and mood disturbances and urinary problems, it has been hypothesized that they are a neuroimaging correlate of age-associated disability (Baezner et al., 2008). The multicenter study Leukoaraiosis and Disability (LADIS) was specifically investigate that patients with severe white matter lesions had more than twice the risk of transition than patients with mild lesions, independently of many other predictors of disability (Inzitari et al., 2007).

Neuropsychiatric symptoms resulting from LA mainly include hallucination, agitation, depression, anxiety, disinhibition, apathy, irritability, sleep disturbance, and appetite change (Tang et al., 2009). 6 There is some evidence that white matter hyperintensities may be related to poor outcomes from delirium, as well as increasing the propensity for delirium (Schmitt et al., 2012). Persons who are depressed when they are older are more likely to have LA to be cognitively impaired and to have an increase in falls (Chen et al., 2015).

Urinary disturbances are common in cerebral vascular pathology, which mainly include nocturia, incontinence, urinary frequency, and urgency (Li et al., 2018). In the LADIS study, Poggesi and colleagues researched 639 individuals with age-related white matter changes (ARWMC) ranging from mild to severe, and reported that 70% of the participants complained of at least one urinary symptom (Poggesi et al., 2008). Urinary urgency is associated with the severity of ARWMC, while urinary frequency is only associated with the stroke history. In patients with Alzheimer's disease, larger ARWMCs in volume were found to be associated with urinary incontinence (Li et al., 2018).

Gait disturbance, characterized by impairment of locomotion, equilibrium and gait ignition, is another common manifestation of LA (Iseki et al., 2010). LA is independently associated with several gait parameters including a lower gait velocity, a shorter stride length and a reduced cadence (de Laat et al., 2010). White matter lesions in the parietal lobe are most highly associated with falls (Murray et al., 2010). The loss of neural connections between the subcortex and the cortex produced by the oxidative damage to the tissue appears to be the major reason for these poor outcomes (Morley, 2015).

## Prognosis

LA is common in the elderly and the prevalence increases with age (Grueter and Schulz, 2012). However, very little is known about the progression and prognosis of this disease, other than its association with age. Currently, only a few studies are available. The Austrian Stroke Prevention Study found that only 17.9% of the study participants had progressive white matter lesions at 3 years of follow-up (Schmidt et al., 1999). In other studies, disease progression was found in 27%, 32%, and 28% of the study subjects at 2-, 3-, and 5-year follow-ups, respectively showing disease progression (Taylor et al., 2003; Van Dijk et al., 2005). A multicenter LADIS study reported finding that 74% of a group of independent elderly patients at baseline had varying degrees of disease progression within 3 years (Inzitari et al., 2009). The risk factors for LA progression appear to be the same as those for the initial onset of disease, namely increasing age and hypertension. The degree of baseline brain damage load may be a prognostic factor, and patients with high brain damage load are more likely to be further impaired (Gouw et al., 2008). Overall, from the limited research data available, the main factors associated with LA progression are advancing age, hypertension, and high baseline lesion load. Clinically, many studies have shown that disease progression in LA is associated with cognitive decline (Cai et al., 2015). The correlation of LA severity with stroke risk and gait impairment remains to be confirmed (Figure 2).

## Conclusion

In summary, the high prevalence and clinical relevance of LA is gaining increasing attention with advances in brain imaging technology. Because age is one of the most important risk factors for this disease, and as the population ages, the prevalence of LA and the consequent prevalence of dementia, mobility impairment, and stroke will increase. Given the impact not only on individuals but also on the health care system, it is critical to understand the risk factors for LA and its prevention and treatment strategies.

## Author contributions

LS and JM contributed to conception and design of this review. LS, LH, and YL organized the database. LS, LH, and XC wrote the first draft of the manuscript. JM and RL reviewed and revised the manuscript. All authors contributed and approved the submitted version.

## Conflict of interest

The authors declare that the research was conducted in the absence of any commercial or financial relationships that could be construed as a potential conflict of interest.

## Publisher's note

All claims expressed in this article are solely those of the authors and do not necessarily represent those of their affiliated organizations, or those of the publisher, the editors and the reviewers. Any product that may be evaluated in this article, or claim that may be made by its manufacturer, is not guaranteed or endorsed by the publisher.
